
JOURNAL INFORMATION
==============================
NLM Title Abbreviation: Behav Neurol
Journal ID: Behav Neurol
NLM Journal ID: 8914585
Journal ID: BN

Behavioural Neurology

ISSN: 0953-4180
EISSN: 1875-8584
Publisher: Wiley

ARTICLE INFORMATION
==============================
PMCID: PMC5434400
PMID: 21422566
DOI: 10.3233/BEN-2010-0306
Article ID: 956709
Article version: 1
Subjects: Research Article

Cultural Factors Influencing Performance on Visuoperceptual Neuropsychological Tasks

Kosmidis Mary H. *
Tsotsi Stella
Karambela Olympia
Takou Eleni
Vlahou Christina H.
School of Psychology Aristotle University of Thessaloniki Thessaloniki Greece
*Mary H. Kosmidis: kosmidis@psy.auth.gr
Print publication date: 2010
Electronic publication date: 2011 Mar 7
Volume: 23
Issue: 4
First page: 245
Last page: 247
Received 2011 Jan 31; Accepted 2011 Jan 31
Copyright: Copyright © 2010 Hindawi Publishing Corporation and the authors.
Copyright year: 2010
License: This is an open access article distributed under the Creative Commons Attribution License, which permits unrestricted use, distribution, and reproduction in any medium, provided the original work is properly cited.
License URL: https://creativecommons.org/licenses/by/3.0/

==============================
Behavioural Neurology 23 (2010) 245–247
DOI 10.3233/BEN-2010-0306
IOS Press

### Cultural factors influencing performance on visuoperceptual neuropsychological tasks

Mary H. Kosmidis*, Stella Tsotsi, Olympia Karambela, Eleni Takou and Christina H. Vlahou
*School of Psychology, Aristotle University of Thessaloniki, Thessaloniki, Greece*

### 1. Introduction

Recent years have seen a growing interest in neuropsychological assessment throughout the world. In countries with no history of neuropsychological research, clinicians often use existing tests developed in other countries with a long tradition in neuropsychological research and practice. While verbal tests were viewed as vulnerable to cultural and/or language bias, visuoperceptual tasks were once considered impervious to cultural factors. More recent studies, however, have dispelled this myth [1]. For example, Colombian Aruacos showed significantly poorer performance on the Rey-Osterrieth copy and immediate memory conditions [2] and Turkish undergraduate students achieved lower scores than an American normative sample on the Group Embedded Figures Test, approaching the task with a unique cognitive style [3]. Even within the same country, ethnic or racial minorities may also be influenced by cultural factors. For example, in the U.S., elderly Spanish-speaking Americans achieved lower scores than English-speaking peers on the Benton Visual Retention Test and Identities and Oddities (Mattis Dementia Rating Scale) [4].

Culture-based influences on neuropsychological performance most likely reflect underlying assumptions regarding not only which functions are worthy of assessment, but also the optimal approach to assessing them. More specifically, implicit beliefs and value judgments may emphasize the importance of particular abilities over others (e.g., verbal vs. interpersonal skills), they ways in which these abilities should manifest (e.g., speedy responding vs. power tests) and the actual nature of the function (e.g., school learning vs. novel problem-solving). In the present investigation, we sought to evaluate the cultural appropriateness of several commonly used neuropsychological tests of visuoperceptual skills and to identify components of these tests that might limit their appropriateness for the Greek population. Additionally, we sought to propose a method for exploring the cultural appropriateness of neuropsychological tests relevant to other countries and ethnic minorities.

### 2. Method

We recruited healthy Greek individuals from the full adult age range from the community to participate in separate studies for the development of normative data. Exclusion criteria were a (self-reported) history of psychiatric or neurological disorder, substance abuse or dependence, head injury or any other medical disorder affecting the central nervous system, based on their self-report. All participants gave written informed consent. The visuospatial tests were administered to each participant individually according to standardized procedure (see Table 1 for the tests administered). We then found normative or lifespan studies conducted in the U.S. or the U.K. to use as comparison groups [5–10]. For these comparisons, we used a subset of each of our samples so as to match them with the foreign samples according to age range.

*Corresponding author: Mary H. Kosmidis, School of Psychology, Aristotle University of Thessaloniki, 54124 Thessaloniki, Greece. Tel.: +30 2310 997308; Fax: +30 2310 997384; E-mail: kosmidis@psy.auth.gr.

ISSN 0953-4180/10/$27.50 © 2010 – IOS Press and the authors. All rights reserved

Table 1

| | Greek | | British | | American | |
|---|---|---|---|---|---|---|
| | Mean | S.D. | Mean | S.D. | Mean | S.D. |
| **VOSP** | ($n = 58$) | | ($n = 160$) | | ($n = 111$) | |
| Silhouettes | 12.7 | 1.8 | 18.8 | 1.4 | 19.5 | 0.7 |
| Incomplete Letters | 16.7* | 4.5 | 22.2 | 4.0 | 20.4 | 3.8 |
| Object Decision | 15.0* | 2.8 | 17.7 | 1.9 | 17.5 | 1.9 |
| Progressive Silhouettes | 11.8* | 2.6 | 10.8 | 2.5 | 9.6 | 2.2 |
| Dot Counting | 9.8 | 0.6 | 9.9 | 0.2 | 9.8 | 0.6 |
| Position Discrimination | 19.5 | 0.8 | 19.6 | 0.9 | 19.5 | 1.3 |
| Number Location | 6.7* | 2.9 | 9.4 | 1.1 | 9.1 | 1.3 |
| Cube Analysis | 7.5* | 2.4 | 9.2 | 1.2 | 9.5 | 0.8 |
| **HVOT** | 18.3* ($n = 88$) | 4.6 | | | 26.2 ($n = 222$) | 2.6 |
| **JLO** | M | W | | | M | W |
| 16–49 years | 27.5* ($n = 42$) 3.1 | 25.0 ($n = 43$) 4.3 | | | 25.6 ($n = 27$) 3.1 | 23.3 ($n = 31$) 4.3 |
| 50–64 years | 25.0 ($n = 20$) 3.7 | 23.8 ($n = 18$) 3.7 | | | 24.3 ($n = 17$) 3.7 | 22.2 ($n = 26$) 3.7 |
| 65–74 years | 24.2 ($n = 10$) 3.2 | 16.7 ($n = 9$) 6.2 | | | 22.7 ($n = 21$) 3.2 | 20.8 ($n = 15$) 6.2 |
| **FR** | | | | | | |
| 60–69 years | 44.6 ($n = 21$) 4.6 | | | | 42.8 ($n = 101$) 4.0 | |
| 70–79 years | 38.2 ($n = 12$) 5.2 | | | | 40.6 ($n = 99$) 4.0 | |
| **R-O** | | | | | | |
| 30–50 years | 34.9* ($n = 105$) 1.6 | | | | 32.8 ($n = 48$) 3.1 | |
| 50–65 years | 33.9* ($n = 88$) 3.5 | | | | 31.2 ($n = 57$) 4.4 | |
| 65–80 years | 29.7 ($n = 45$) 6.2 | | | | 31.4 ($n = 102$) 4.0 | |
| **GEFT** | 14.1* ($n = 58$) 4.3 | | | | 12.0 ($n = 70$) 4.6 | |

### 3. Results

Results differed based on each test. Our Greek samples performed more poorly than both comparison groups on most subtests of the (VOSPT) subtests. In particular, Greek adults performed more poorly on Silhouettes [Greek vs. British: $t(216) = 8.653$, $p < 0.05$; Greek vs. American: $t(167) = 5.641$, $p < 0.05$], Object Decision [Greek vs. British: $t$ (216) = 8.13, $p < 0.05$; Greek vs. American: $t(167) = 7.02$, $p < 0.05$], Progressive Silhouettes [Greek vs. British: $t(216) = 2.55$, $p < 0.05$; Greek vs. American: $t(167) = 5.70$, $p < 0.05$], Number Location [Greek vs. British: $t(216) = 9.89$, $p < 0.05$; Greek vs. American: $t(167) = 7.26$, $p < 0.05$], and Cube Analysis [Greek vs. British: $t(216) = 6.87$, $p < 0.05$; Greek vs. American: $t(167) = 2.97$, $p < 0.05$], but not on Dot Counting and Position Discrimination. The Greek sample also performed worse than the comparison group on the Hooper Visual Organization Test (HVOT) [$t(308) = 19.06$, $p < 0.05$]. No group differences emerged on the Judgement of Line Orientation (JLO), with the exception of better performance of the Greek, relative to the American, young male group (age group 16–49 years) [$t(67) = 2.48$, $p < 0.05$]. Performance was also similar between the two samples on the Face Recognition (FR) [age group 60–69: $t(120) = 1.90$, $p > 0.05$; aged 70–79 $t(109) = 1.92$, $p > 0.05$]. Finally, the group difference on the (R-O) copy [$t(392) = 9.80$, $p > 0.05$] and the Group Embedded Fiqures Test (GEFT) [$t(126) = 2.65$, $p > 0.05$] favored the Greek over the American sample.

### 4. Discussion

The findings of the current study indicated poorer performance of the Greek sample as compared with published studies from the U.S. and the U.K. on some visuospatial tasks, namely the VOSPT and the HVOT, but similar performance on others, such as the JLO and the FR, with a definite Greek advantage on the R-O and the GEFT. We propose that tests requiring a certain level of abstraction in order for an examinee to respond correctly (i.e., VOSPT, HVOT) may yield lower scores in the Greek sample when compared with samples from studies in other countries. In contrast, tests calling for more concrete perceptual skills yielded similar responses between Greek and foreign samples. Finally, better performance on tests requiring the processing of geometric shapes may reflect the extensive exposure to geometry in the Greek as compared to the American school system.

Limitations to the generalizability of our findings emerge from the post hoc nature of our study, as well as the lack of data from the full adult range and information on educational level in some of the comparison samples. Our next step is to explore the ability of these tests to correctly classify patients and healthy individuals in order to establish their clinical utility for the Greek population.

Our current findings highlight the importance of exploring the component features of existing tests in order to exclude features that may complicate the assessment of a particular skill in a given cultural group, instead of merely developing norms for existing foreign tests. This process may aide in the design and development of new, culture-appropriate tests. We believe that the cultural appropriateness of specific neuropsychological tests remains an empirical question.

### Acknowledgement

The present study was supported by a Marie Curie International Reintegration Grant (7th Framework Programme)

### References

[1] I. Herrera-Guzmán, J. Peña-Casanova, J.P. Lara, E. Gudayol-Ferré and P. Böhm, Influence of Age, Sex, and Education on the Visual Object and Space Perception Battery (VOSP) In a Healthy Normal Elderly Population, *The Clinical Neuropsychologist* **18** (2004), 385–394.

[2] M. Rosselli and A. Ardila, The impact of culture and education on non-verbal neuropsychological measurements: a critical review, *Brain and Cognition* **52** (2003), 326–333.

[3] M. Cakan, Psychometric data on the Group Embedded Fibures Test for Turkish undergraduate students, *Perceptual and Motor Skills* **96** (2003), 993–1004.

[4] D.M. Jacobs, M. Sano, S. Albert, P. Schofield, G. Dooneief and Y. Stern, Cross-cultural neuropsychological assessment: a comparison of randomly selected, demographically matched cohorts of English- and Spanish-speaking older adults, *Journal of Clinical and Experimental Neuropsychology* **19** (1997), 331–339.

[5] A. L. Benton, A. B. Sivan, K. deS. Hamsher, N.R. Varney and O. Spreen, *Contributions to Neuropsychological Assessment. A clinical manual* (2nd ed.), Oxford University Press, New York, 1994.

[6] P.J. Bonello, L.J. Rapport and S.R. Millis, Psychometric Properties of the Visual Object and Space Perception Battery in Normal Older Adults, *The Clinical Neuropsychologist* **11** (1997), 436–442.

[7] K.J. Christensen, B.E. Riley, K.A. Heffernan, S.B. Love and M.E. McLaughlin Sta. Maria, Facial recognition test in the elderly: norms, reliability and premorbid estimation, *The Clinical Neuropsychologist* **16** (2002), 51–56.

[8] A.L. Cummings and H.G. Murray, Psychometric data on the Group Embedded Figures Test for a sample of adult learners, *Perceptual and Motor Skills* **65** (1987), 583–586.

[9] P.S. Fastenau, N.L. Denburg and B.J. Hufford,. Adult norms for the Rey-Osterrieth Complex Figure Test and for Supplemental Recognition and Matching Trials from the Extended Complex Figure Test, *The Clinical Neuropsychologist* **13** (1999), 30–47.

[10] A.L. Jefferson, S. Wong, E. Bolen, A. Ozonoff, R.C. Green and R.A. Stern, Cognitive correlates of HVOT performance differ between individuals with mild cognitive impairment and normal controls, *Archives of Clinical Neuropsychology* **21** (2006), 405–412.
